# Characterization, Preparation, and Purification of Marine Bioactive Peptides

**DOI:** 10.1155/2017/9746720

**Published:** 2017-07-06

**Authors:** Xueqin Wang, Huahua Yu, Ronge Xing, Pengcheng Li

**Affiliations:** Institute of Oceanology, Chinese Academy of Sciences, Qingdao 266071, China

## Abstract

Marine bioactive peptides, as a source of unique bioactive compounds, are the focus of current research. They exert various biological roles, some of the most crucial of which are antioxidant activity, antimicrobial activity, anticancer activity, antihypertensive activity, anti-inflammatory activity, and so forth, and specific characteristics of the bioactivities are described. This review also describes various manufacturing techniques for marine bioactive peptides using organic synthesis, microwave assisted extraction, chemical hydrolysis, and enzymes hydrolysis. Finally, purification of marine bioactive peptides is described, including gel or size exclusion chromatography, ion-exchange column chromatography, and reversed-phase high-performance liquid chromatography, which are aimed at finding a fast, simple, and effective method to obtain the target peptides.

## 1. Introduction

The oceans occupy more than 70% of the earth and are a rich natural resource for many bioactive compounds in organisms such as fish, shellfish, molluscs, univalves, cephalopods, crustaceans, and echinoderms, which significantly contribute to economic and research development [1, 2]. Since marine organisms live in complex habitats and are exposed to extreme conditions, such as salinity, pressure, temperature, and illumination, they produce a wide variety of secondary metabolites that cannot be found elsewhere [2]. In addition, the marine organisms also have special structures and constitute nearly half of the worldwide biodiversity, like antioxidant activity, antimicrobial activity, anticancer activity, antihypertensive activity, anti-inflammatory activity, and so forth [3].

In general, bioactive peptides often have 3 to 20 amino acid residues, and their biological activities are based on their amino acid composition and sequence [4]. Recently, much attention has been paid to unravelling the structural, compositional, and sequential properties of bioactive peptides [3]. This review highlights the characteristics of marine peptides with biological activities as well as the preparation and purification of such peptides.

## 2. Marine Peptides with Different Bioactivities

Many marine organisms are exposed to more extreme conditions than that on land, which make the marine bioactive peptides have significant different amino acid compositions and sequences from land bioactive peptides; besides, the species and amounts of marine bioactive peptides are more than that of land bioactive peptides. Moreover, Marine bioactive peptides can be obtained from various marine animals, plants, and lower organisms. Each is unique as a species, considering its great taxonomic diversity and special characteristics, marine bioactive peptides have better bioactivity in some areas than land bioactive peptides.

### 2.1. Antioxidative Peptides

Oxidation is an essential reaction in all living organisms, as the formation of free radicals and other reactive oxygen species (ROS) plays an important role in signal transduction [5]. However, excess free radicals can cause many human diseases, such as heart disease, strokes, arteriosclerosis, diabetes, and cancer [6]. Antioxidants are compounds that can inhibit oxygen-dependent lipid oxidation, usually by scavenging and thereby neutralizing free radicals [7]. In addition, the synthetic antioxidants such as butylated hydroxyanisole (BHA) and butylated hydroxytoluene (BHT) have long-term safety problems and negative consumer perception [8]. For these reasons, the demand for natural antioxidants has increased recently.

Compared to the earth environment, marine organisms live in complex habitats and are exposed to extreme conditions; thus, some of them have higher antioxidant activities. In recent years, many antioxidative peptides from marine organisms have been found, such as those from Hoki* (Johnius belengerii)* frame [9], Mackerel* (Pneumatophorus japonicus)* [10], Mussel* (Perna canaliculus)* muscle [11], Croaker* (Otolithes ruber)* [12], Tuna backbone [13], and Prawn* (Penaeus japonicus)* [14], and these peptides show significant free radical scavenging activities (Table 1). Moreover, every year a considerable amount of total catch is discarded [15], causing environmental pollution and the wasting of resources. Therefore, many researchers used seafood by-products to prepare antioxidative peptides, like Sardinelle* (Sardinella aurita)* by-products [16], Abalone* (Haliotis discus hannai Ino)* viscera [17], Nile Tilapia skin [18], Jumbo Squid* (Dosidicus gigas)* skin [19], and so forth, and, thus, these studies were increasing the utilization value of marine organisms.

The measurement of antioxidant activity is an important screening method. Some chemical methods are used, including reducing power, hydroxyl radical scavenging activity, superoxide anion radicals scavenging activity, scavenging reactive oxygen species, and inhibition of lipid peroxidation [20–23]. Despite the wide use of these chemical antioxidant activity assays, none of them take into account the bioavailability, uptake, and mechanism of the antioxidant compounds [24]. In recent years, cell culture models provide an approach that is cost-effective and relatively fast and can explain metabolic issues [25]. One approach is to use the cellular antioxidant status measured by the methyl thiazolyl tetrazolium assay and protect HepG2 cells against H_2_O_2_-induced cytotoxicity [26, 27]. However, since the concentration of H_2_O_2_ is not clear, this method should have a preliminary experiment. Another effective cellular antioxidant activity (CAA) assay is also related to HepG2 cells [25, 28], and the CAA assay is considered a superior indicator of* in vivo* activity compared with* in vitro* assays because it involves the exposure of the antioxidants to the complexity of biological substrates under physiological conditions [29]. Certainly, the best antioxidant assays are from animal models and human studies [30], but they are expensive, time-consuming, and not suitable for the initial screening [24]. In other words, although there is a great multiplicity of methods used for antioxidant testing, there are no approved standardized methods.

### 2.2. Angiotensin-I-Converting Enzyme (ACE) Inhibitory Peptides

Hypertension is one of the most common cardiovascular diseases worldwide [54]. Approximately 54% of strokes, 47% of ischaemic heart disease, 75% of hypertensive disease, and 25% of other cardiovascular diseases worldwide were attributable to high blood pressure [55]. Among the processes related to hypertension, Angiotensin-I-Converting Enzyme (ACE) plays an important role in the regulation of blood pressure. ACE can catalyse the conversion of angiotensin I to angiotensin II, and angiotensin II is a potent vasoconstrictor that increases peripheral vascular resistance and consequently elevates arterial pressure [56, 57]. Therefore, in the development of drugs to control high blood pressure, ACE inhibitors and angiotensin receptor blockers are now used clinically for the treatment of various cardiovascular diseases [58]. However, the synthetic drugs such as captopril, lisinopril, and enalapril [59] are believed to have certain side effects such as a cough, skin rash, loss of taste, or angioneurotic oedema [60, 61]. Due to these adverse side effects, there is a trend towards encouraging the development of natural ACE inhibitors.

In recent years, naturally occurring peptides with ACE inhibitory activity were obtained from various marine organisms such as Green Algae [62], Sea Cucumber* (Acaudina molpadioides)* [31], Tuna [32], Sole* (Limanda aspera)* [33], Blue Mussels* (Mytilus edulis)* [34], Jumbo Squid* (Dosidicus gigas)* [63], Oysters* (Crassostrea gigas)* [64], and Shrimp [35, 36]. In addition, fish are sources of numerous bioactive peptides with ACE inhibitory activities including Alaska Pollack* (Theragra chalcogramma)* frame [37] and skin [38], Flounder fish* (Paralichthys olivaceus)* [65], Tuna [32], Shark [39], and Cod* (Gadus morhua)* [66]. Marine organisms may become important protein resources for the selection of novel ACE inhibitors (Table 1).

To date, the most commonly used method for the detection of ACE inhibitory activity is evaluated by Lineweaver-Burk plots [56]. Additionally, there are many methods for evaluating the ACE inhibitory activity* in vitro*, such as spectrophotometric, fluorometric, radiochemical, high-performance liquid chromatography (HPLC) and capillary electrophoresis (CE) methods [67, 68]. However, the spectrophotometric assay is complicated and time-consuming; the fluorometric assay is expensive but easy and automated [69]; and the radiochemical assay is unsafe and time-consuming and require special apparatus [70]. The HPLC assay has a high sensitivity and short operation time, while in comparison to the methods mentioned above, the CE assay is found to be faster and more automated and requires less sample, substrates, and reagents, which suggests that the CE method is more suitable for the high throughput screening of peptides with ACE inhibitory activity [54]. In addition, there is not necessarily an* in vivo* effect after the identification of an ACE inhibitory peptide* in vitro*. Thus it is necessary to perform* in vivo* animal studies using animal models, and the* in vivo* assay of ACE inhibitory activity is generally conducted by measuring the blood pressure response in spontaneously hypertensive rats following intravenous injection or oral administration [71]. However, the* in vivo* assays are expensive, time-consuming, and complicated. In conclusion, the establishment of a simple, rapid, sensitive, and reliable inhibition assay is desirable.

### 2.3. Antimicrobial Peptides

The discovery of the widespread distribution of antimicrobial peptides (AMPs) over the past 20 years has provided insights into the innate defence systems that permit multicellular organisms [72], and AMPs are considered as highly significant immune effectors that have evolved through positive selection [73]. Recently, much attention has been paid to marine-derived bioactive peptides due to their special living environment, compositions, and properties. The marine organisms are in close contact with microbes and provide a huge source of AMPs. In addition, open ocean seawater harbours have 106 bacterial and 103 fungal cells per millilitre, and most marine organisms host specific populations of microbes on their surfaces or within the confines of their tissues [74]. As stated earlier, this section is to introduce several marine-derived natural products that possess significant antimicrobial properties. In recent years, researchers have isolated AMPs from Atlantic Cod* (Gadus morhua)* [75], Mud Crab* (Scylla paramamosain)* [76], Oyster* (Crassostrea gigas)* [40], Yellow Catfish* (Pelteobagrus fulvidraco)* [41], Sponge* (Trichoderma sp.)* [77], and Marine Snail* (Cenchritis muricatus)* [42], and the AMPs from marine organisms have safe, natural, inexpensive, and high bioactivity properties (Table 1). In addition, several methods for testing the antimicrobial activity of hydrolysates or peptides have been used. For example, the agar diffusion assay is a common method used to test the antimicrobial activity of peptides [78, 79]. This method quantifies the ability of antibiotics to inhibit bacterial growth [80]. The agar diffusion technique is usually used for determining the minimum inhibitory concentration in solid media [81]. Furthermore, there are some other assays to evaluate the antimicrobial activity like the disc diffusion assay [82], broth dilution [83], high throughput fluorescence screening assay [84], and so forth. The growing problem of resistance to conventional antibiotics and the need for new antibiotics has stimulated interest in the development of antimicrobial peptides as human therapeutics [72].

### 2.4. Other Bioactive Peptides

The peptides from marine organisms also exhibit other bioactivities, such as calcium binding, anticoagulant, antitumour, cardiovascular protective, immunomodulatory, neuroprotective, antidiabetic, and appetite suppression activities [85, 86].

There are many researches about the above biological activities; for example, Jung and Kim [43] prepared a peptide from Hoki* (Johnius belengerii)* bone showing significant Ca-binding activity, and the bone could be used in nutraceuticals with a high bioavailability of calcium. Jung et al. [44] also found a low molecular weight peptide with a high affinity to calcium from Alaska Pollack* (Theragra chalcogramma)* backbone, which makes it possible to utilize the fish backbone in the nutraceutical field. Furthermore, with cardiovascular disease being identified as the leading cause of death worldwide, some researchers have separated cardiovascular protective peptides from Yellowfin Sole* (Limanda aspera)* [45], Spirulina Maxima [46], Blue Mussel* (Mytilus edulis)* [47], and other marine organisms (Table 1).

In recent years, there is also a trend to focus on marine organism protein hydrolysates that are used as antitumour agents [87]. For example, Wang et al. [48] have studied the antitumour activity of the Oyster* (Crassostrea gigas)* hydrolysates in BALB/c mice and found the spleen proliferation of lymphocytes and the phagocytic rate of macrophages in S180-bearing mice significantly increased after the administration of the oyster hydrolysates. Hsu et al. [49] have investigated the antiproliferative activities of peptides from Tuna Dark* (Thunnus tonggol)* muscle by-product, and the results showed that the peptide fraction with the molecular weight range from 390 to 1400 Da possessed the greatest antiproliferative activity. Alemán et al. [88] have proven that giant squid gelatine hydrolysates demonstrated an* in vitro* cytotoxic effect on cancer cells, with IC50 values of 0.13 and 0.10 mg/mL for MCF-7 (human breast carcinoma) and U87 (glioma) cell lines, respectively. In addition, the effect of immunomodulatory peptides may be due to enhanced macrophage activity and lymphocyte proliferation. Some researchers have found that the phagocytic activity of peritoneal macrophages is enhanced following the administration of fish protein concentrate from Pacific Whiting* (Merluccius productus)* at 0.3 mg/ml for 7 days [50]. Yang et al. [89] studied the immunomodulatory effects of marine oligopeptide from Chum Salmon hydrolysate, and, in comparison with the control group, the salmon hydrolysate could significantly enhance the capacity for lymphocyte proliferation. Furthermore, as part of our innate immune system, inflammation is one of the most generic responses, but uncontrolled inflammation is believed to play crucial roles in the pathogenesis of various diseases [90], and there has been a remarkable increase in pharmacological research on anti-inflammatory marine biomolecules in recent years. Novel bioactive peptides from sponges [91], Algae* (Pyropia yezoensis)* [51], Brown Seaweed [92], and Abalone [93] are also described along with their pharmacological effects in relation to anti-inflammation.

Furthermore, some other bioactive peptides from marine organisms have been investigated. Lee et al. [94] have investigated the antidiabetic effect and mechanism of a Marine Algae (*I. foliacea*) product in C57BL/KsJ-db/db mice and found that the levels of postprandial blood glucose were significantly lower than the control group. Zhu et al. [52] have also found that the oligopeptides from Marine Salmo* (Oncorhynchus keta)* skin could significantly reduce the fasting blood glucose in diabetic rats, and they concluded that the antidiabetic activity may be mediated by downregulating T2DM-related oxidative stress and inflammation. Moreover, as obesity has become a serious public health problem throughout the entire world, some marine peptides were found to have antiobesity activity, such as Seaweeds [95], Cod [96], Blue Whiting* (Micromesistius poutassou)*, Brown Shrimp* (Penaeus aztecus)*  [53], and other marine organisms [97]. Although marine organisms comprise roughly one-half of the total global biodiversity and a number of studies exist for proving the biological effects using* in vitro* experiments or animal models, it is now important to use human intervention trials to study the biological effects and their mechanisms in more detail [86, 98].

## 3. Preparation of Marine Bioactive Peptides

The bioactive peptides were different depending on their species, amino acid composition, and sequence, and they can prepared by different methods. Moreover, certain methods also affect the biological activities of peptides [99].

### 3.1. Organic Synthesis

With the development of technologies and methodologies for structural elucidation, organic synthesis is increasingly applied to marine natural products [100]. Due to their special bioactivities, marine natural products have yielded a considerable number of drug candidates, ranging from simple peptides to cyclic peptides, and organic synthesis is always used to batch synthetic target peptides due to the purification production being low [101]. Organic synthesis usually chooses a solid-phase synthesis method using a series of solvents and synthesis methods to obtain the target peptides, and the coarse product is identified by mass spectrometry to test whether it is consistent with the theoretical molecular weight. Its further biological activity would also be verified. Organic synthesis would realize high-volume production of the target peptides. However, the organic synthesis technique is time-consuming, expensive, and environmentally unfriendly. This technique also requires target peptides with a clear sequence. Then the researchers should identify the compositions of peptides using a series of isolation and purification technologies, and, thus, better extraction techniques are preferred.

### 3.2. Microwave Assisted Extraction

In the last decade, microwave assisted extraction has been successfully applied for the extraction of numerous biologically active compounds from a wide variety of natural resources [102, 103]. This technique involves the use of electromagnetic radiation in a frequency ranging from 300 MHz to 300 GHz to heat solvents in contact with a sample to separate compounds of interest from the sample matrix [104]. This technique has been reported to enhance the extraction yield of bioactive compounds from various matrices compared to traditional solid-liquid extraction [105]. The mechanism of microwave assisted extraction is through inter- and intramolecular friction, together with the movement and collision of a very large number of charge ions, causing the rapid heating of the reaction system and resulting in the breakdown of cell walls as well as membranes [106]. Although the use of microwave assisted extraction may degrade bioactive carbohydrates due to the localized high temperature [107], there are many reports about extracting bioactive materials from marine organisms using microwave assisted extraction. For example, some researchers have applied a microwave assisted extraction method for fish tissues [108], Oysters [109], and Shrimp [110], and microwave assisted acid hydrolysis of proteins for peptide mass mapping and tandem mass spectrometric analysis of peptides has been reported [111].

Additionally, the microwave assisted technology is suitable for degrading the special organisms, such as Algae, that have cells that are surrounded by a dynamic, complex, and carbohydrate-rich cell wall, which makes the breakdown of cell walls particularly important [112]. For example, some researchers have studied the antioxidant capacity of sulphated polysaccharides from Brown Seaweed [113, 114] using microwave assisted extraction under different pressures, extraction times, and algae/water ratios, and these studies indicated that microwave assisted extraction was an effective technology. Moreover, mechanical disruption techniques are also very useful to break down calcareous and siliceous skeletons of some hard sponges [106].

In general, the compounds are extracted more selectively and quicker by this technique, with similar or better yields in comparison with conventional extraction processes. Meanwhile, this technique also uses less energy and solvent volume, has reduced costs, and is more environmentally friendly than traditional extraction processes [115].

### 3.3. Chemical Hydrolysis

Chemical hydrolysis of proteins is achieved by cleaving peptide bonds with either acid or alkaline. This method has been widely used in the past for the industry because it is inexpensive and quiet simple to conduct. However, this technology has many limiting factors such as it being a difficult process to control and trend to give modified amino acids [98] and yielding products with variable chemical compositions and functional properties. Acid hydrolysis is an important chemical modification that can significantly change the structure and functional properties of peptides [116]. Acid hydrolysis is preferred over other pretreatments because of its low cost and effectiveness [117]. The most common type of dilute acid used is sulfuric acid (H_2_SO_4_). However, nitric acid (HNO_3_), hydrochloric acid (HCl), phosphoric acid (H_3_PO_4_), and other acids have also been investigated [118]. Interestingly, maleic acid and oxalic acid were more efficient in biomass hydrolysis than a dose of H_2_SO_4_ [116]. The acid hydrolysis of fish scales has usually involved HCl [119], and other fish, such as scup, salmon, bluefish, and Mackerel, were hydrolysed by 25% of 0.4 M HCl [120]. However, acid hydrolysis usually requires high temperature, and the hydrolysate contains a large amount of salt. Furthermore, acid hydrolysis could destruct the tryptophan, which is an essential amino acid [121]. On the other hand, there are some researches about alkali hydrolysis on samples like Cod [122], Tilapia [123], Channel Catfish [124], and so forth, but alkali hydrolysis often results in poor functionality and low nutritive value [121]. Furthermore, desalination in the later experiment is also complex. Additionally, high collagen solubility is also observed with alkali treatment [125–127]. In other words, chemical hydrolysis can easily cause peptide bond hydrolysis and obtain a high yield of peptides, but this technology is insecure and environmental unfriendly, thus making it mainly used for industrial production.

### 3.4. Enzyme Hydrolysis

Enzymatic modification of proteins using selected proteolytic enzyme preparations to cleave specific peptide bonds is widely used in the food industry [128]. Enzymatic proteolysis from animal and plant sources has been studied extensively and described by several different authors over the last 60 years [121], and it is still the most commonly used method for adding value to the target organism. The preferred commercial enzymes are prepared from bacterial origin, including Alcalase [13, 129], Neutrase [130, 131], and Flavourzyme [132, 133], as well as from animals and plants, including trypsin [68, 134], Pepsin [135, 136], Papain [137, 138], Bromelain [139, 140], and Subtilisin [141, 142]. Furthermore, the addition of exogenous enzymes could make the hydrolytic process more controllable and reproducible. There are five independent variables of enzyme hydrolysis including the following: enzyme concentration, pH, extraction temperature, extraction time, and water/material ratio, with each enzyme having different hydrolysis conditions [143]. For example, Bhaskar et al. [144] used Alcalase with the optimum conditions of an enzyme to substrate level of 1.5%, and a hydrolysis time of 135 min to hydrolyse visceral waste proteins of Catla* (Catla catla)* and obtain a higher degree of hydrolysis close to 50%. Another researcher used Protamex with the optimum conditions of an enzyme to substrate level of 4%, a pH of 7.1 and a temperature of 51°C to hydrolyse Blue Shake skin in order to obtain peptide with the highest degree of hydrolysis [145]. In addition, Song et al. [146] studied the hydrolysis conditions of Pepsin with an enzyme to substrate level of 1100 U/g, a pH of 2.0, a reaction time of 2.4 h, and a water-to-substrate ratio of 4 : 1 (v/w). In a word, there are many researchers who have focused on the enzyme hydrolysis due to its reproducibility and controllability [147]; besides, enzymatic reactions do not involve side reactions and do not reduce the nutritional value of the protein source. However, adjusting the pH with acid or alkali may add inorganic mass, such as salt, which may be difficult and costly to remove later in the process.

## 4. Purification of Marine Bioactive Peptides

The peptides usually have 3–20 amino acid residues, and their bioactivities are based on their amino acid compositions and sequences. Recent studies have shown that most peptide sequences encrypted in food proteins confer bioactive properties after release by enzymatic hydrolysis [148]. Then it is important to identify the peptide structure and that is why so many researchers have investigated the peptide purification.

**Figure 1:**
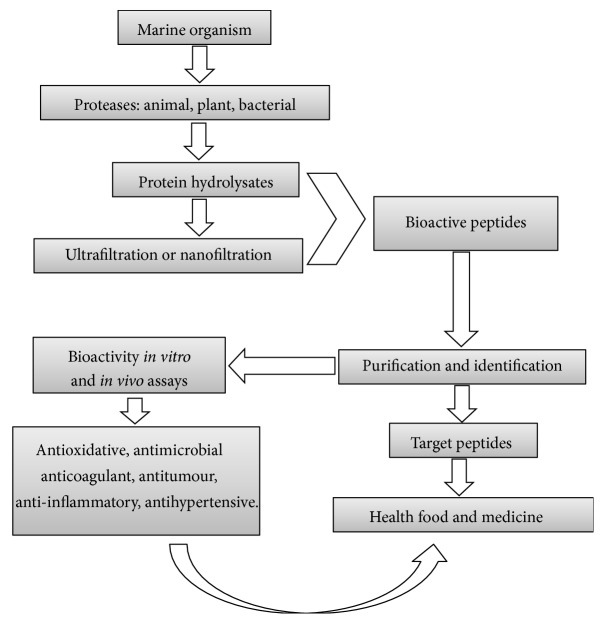
Schematic diagram for preparation and purification of biological peptides.

In a typical procedure for discovery of marine bioactive peptides, the peptides firstly extracted from the marine organisms, the extract is screened for a special bioactivity, fractionated using a bioassay-guided fractionation technology, and finally purified to yield a single bioactive peptide. In addition, to develop an efficient purification process, it is necessary to clearly research methods such as membrane filtration systems, gel or size exclusion chromatography, ion-exchange column chromatography, and reversed-phase high-performance liquid chromatography (RP-HPLC) (Figure 1). Each purification technology has its own advantages and disadvantages, which the researcher should consider clearly before the purification of peptides.

### 4.1. Membrane Filtration

**Figure 2:**
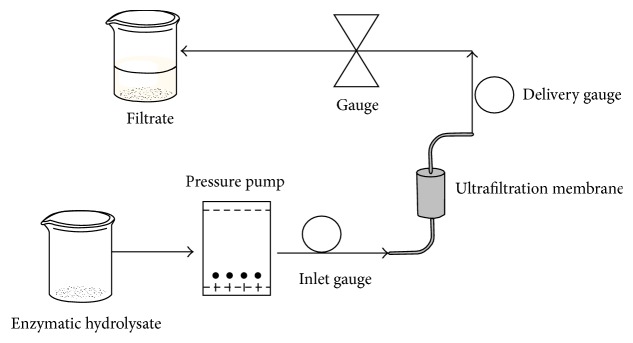
The flow chart of ultrafiltration method.

Advances in material science and membrane manufacturing technology have made the membrane technique grow to be an important technology for the separation of natural products [149]. Generally speaking, to obtain the target peptide, the initial peptide is usually separated by membrane filtration first. Membrane filtration can be used at different levels. Ultrafiltration with a high molecular weight cut-off (MWCO) can be used for the separation of macropeptides and nonhydrolyzed proteins. In normal conditions, the peptides have 3–20 amino acid residues, and membranes with an MWCO at 1–10 kDa are suitable for the fractionation of bioactive peptides with desired molecular weights. Membranes with a low MWCO at approximately <1 kDa are used to concentrate the peptides. Furthermore, membrane filtration can operate at normal temperature, and there are no chemical reactions during the process (Figure 2). Membrane filtration can provide a large number of separation compared to other chromatographic separation, and then this technology always shows applications for the separation and recovery of bioactive compounds from diverse raw matrices. However, membrane filtration is restricted to desalination due to the poor selectivity of the membrane, while most of chromatographic separation could desalinize, and some researchers have used a nanofiltration membrane for desalination [150]. Certainly, active carbon is also used for desalination [151].

In recent years, many researchers have used membrane filtration as the first purification step. For example, Cho et al. [152] used cross-flow microfiltration to make the galacturonic acid content of pectin increase from 68.0 to 72.2%. Kim et al. [9] used ultrafiltration membranes to separate the Hoki* (Johnius belengerii)* frame protein hydrolysates (HPH) and found that HPH-III with a molecular weight distribution of 3–5 kDa showed the highest antioxidant activity. Moreover, with ultrafiltration, Wang et al. [10] concentrated and prepurified antioxidative peptides extracted from Mackerel, and they found that the peptide with molecular weight of below 3 kDa displayed the highest 1,1-diphenyl-2-picrylhydrazyl radical scavenging activity. Tonon et al. [153] have obtained a protein hydrolysate from Shrimp by coupling ultrafiltration, and Roblet et al. [154] have used electrodialysis with filtration membranes to purify Atlantic salmon frame protein hydrolysate.

In summary, membrane filtration technology has demonstrated potential application in the separation of bioactive products. The main problem with membrane separation is fouling, which could shorten the membrane life and increase cost. As a result, modification of the structure and properties of the membrane and the development of new membrane systems with low fouling characteristics and high selectivity would promote the development of membrane filtration technology.

### 4.2. Gel Filtration Chromatography

The partially purified extract is subjected to gel filtration chromatography and ion-exchange chromatography, with reversed phase C_18_ HPLC used in the final purification step [155, 156].

Gel filtration chromatography (GFC), also called size exclusion chromatography, has been employed for over 40 years for the separation, desalting, and molecular weight estimation of peptides and proteins. GFC is the simplest and mildest of all of the chromatography techniques and separates molecules on the basis of differences in size. Its separation mechanism is to filter molecules according to their sizes; some smaller molecules enter the pores of the gel and travel a longer distance, while larger molecules show much shorter retention times. Unlike ion-exchange chromatography and others, molecules do not bind to the chromatography medium so the buffer composition does not directly affect the resolution. Consequently, a significant advantage of GFC is that elution conditions can be varied to suit the type of sample as well as the requirements for further purification, analysis, or storage without altering the separation. GFC is well-suited to biomolecules that are sensitive to changes in pH, concentrations of metal ions, or cofactors as well as harsh environmental conditions and can be used directly after ion-exchange chromatography since the buffer composition will not generally affect the final separation. In addition, GFC has high selectivity and high resolution, which is an important step in a purification scheme.

The GFC also has some limitations, such as the loading amount being seldom compared to the membrane filtration and collecting sample costing a lot of time. In addition, the resolution is influenced by many factors, like the particle size, particle uniformity, bed height, column packing quality, flow rate, sample concentration and volume, and so forth. The molecular weight range over which a GFC medium can separate molecules is referred to as the selectivity of the medium. Today's GFC media cover a molecular weight range from 100 to 8 × 10^7^ Da, separating biomolecules from peptides to very large proteins and protein complexes. There are many GFC media and different media have special properties. For example, Superdex Increase or Superdex is designed for high resolution, short run times, and high recovery. Huang et al. [157] found a novel polysaccharide peptide with a molecular weight of 9.17 × 10^4^ Da that was obtained from* Clinacanthus nutans Lindau* leaves through purification with Superdex 200 and DEAE Sepharose Fast Flow. Qian et al. [158] also used Superdex 200 to purify a protein with a molecular weight of 4.3 × 10^4^ Da, and Pan et al. [159] purified the fish scale-degrading enzyme with molecular weight of 1.19 × 10^5^ Da. Superdex prep grade [160, 161] and Sephacryl [162, 163] are suitable for fast, high-recovery separation at laboratory and industrial scales. For example, Wu et al. [164] purified trypsin inhibitor from Yellowfin Tuna* (Thunnus Albacores)* roe, followed by column chromatography on Sephacry S-200, Sephadex G-50, and DEAE-cellulose, and it was finally found to have an apparent molecular weight of 7 × 10^4^ Da. In addition, Sephadex is recommended for rapid group separation such as desalting and buffer exchange and it is widely used in the marine organism purification field [16, 165]. For example, Jai ganesh et al. [156] and Vijaykrishnaraj et al. [11] used Sephadex G-25 to separate* Parastromateus Niger* viscera and mussel flavour, respectively. In addition, Ma et al. [166] performed the study on the purification of Marine Yeast using Sephadex G-75.

In other words, although GFC is cumbersome, time-consuming, and costly, its high selectivity and high resolution make this technology applicable to various separation and purification fields.

### 4.3. Ion-Exchange Chromatography

In recent years, the utilization of ion-exchange chromatography (IEX) techniques for the separation, detection, and structural determination of proteins, peptides, and small nucleotides has become increasingly important [167]. IEX media have charged functional groups that bind molecules with an opposite charge. Bound molecules are eluted from the medium by displacement, via the application of an increasing concentration of a similarly charged molecule. Proteins have numerous functional groups that can have either positive or negative charges. By adjusting the pH or the ionic concentration of the mobile phase, proteins can be separated. IEX is used for capturing the target protein or bulk impurities from large-volumes, as an intermediate purification step or as a final step for high resolution purification to remove impurities.

Since ion exchange is an adsorption technique, it can be used in either positive or negative capture modes. Depending on the pH or conductivity of the sample, the target may adsorb while the contaminant is unretained, and this is referred to as positive chromatography. In reverse, it is referred to as negative chromatography. In addition, there is an extensive range of IEX media and a suitable IEX medium can be chosen depending on the target, sample, and resolution that are needed. The media include Capto, MacroCap, MiniBeads, MonoBeads, Sephadex, Sepharose, and SOURCE. Each media has its special working pH, buffer system, and capacity, and, thus, it is used for purifying different type of samples. We can use Sephadex media as an example. DEAE Sephadex is a weak anion exchanger, and its working pH is 2–9; QAE Sephadex is a strong anion exchanger, and its working pH is 2–12; and CM Sephadex and SP-Sephadex are weak cation and strong cation exchangers, respectively, and their working pH ranges are 6–10 and 4–13, respectively.

Based on above-mentioned properties, many researchers have used different IEX media to purify the target product. For example, Li et al. [151] have used CM Sephadex C-25 to separate chitooligomers with the elution requirement of HAc–NaAc buffer (50 mM, pH = 4.8) and different concentrations of NaCl (0–2 M)–HAc buffer stepwise at 3 mL/min. Park et al. [168] have purified the antioxidant peptide from Blue Mussel* (Mytilus edulis)* hydrolysate with SP-Sephadex C-25 cation exchanger, which was equilibrated with 50 mM sodium acetate buffer (pH = 4.0). In addition, SP-Sephadex C-25 is used by other researchers to purify the target peptide [169, 170]. Additionally, other media such as CM Sepharose Fast Flow [171, 172], DEAE Sepharose Fast Flow [173–175], SP Sepharose Fast Flow [176, 177], Q Sepharose Fast Flow [178], and so forth were applied to purify marine organisms. The great advantage of IEX is the implementation of mass separation compared to GFC, which could save time and improve accuracy. However, IEX is also costly, complex, and is not well-suited to biomolecules that are sensitive to pH, metal ions, and other factors. Further research on IEX may focus on finding a cheap and high resolution material to replace the expensive media. Although this technology is difficult to realize, IEX will be widely applied in biological separation in the future.

### 4.4. High-Performance Liquid Chromatography

HPLC is the most widely used technique for the separation, identification, and purification of bioactive peptides [179]. Analysis HPLC could fully reflect the information of the sample and do not need to collect fractions; preparative HPLC need to consider the purity, production, production cycle, and operating cost. In addition, RP-HPLC can be used to fractionate peptides based on their hydrophobic properties, especially when studying the structural and functional properties of peptides [180, 181]. The main advantages of this technology include the ease of operation, high resolution, and sensitivity, and it always uses a short time to get the elution spectra compared to the GFC and IEX, which always need twenty to thirty hours. In recent years, there are many researchers that have used HPLC to purify marine organisms, like Enteromorpha [182], Cyanobacterium [183], Thornback Ray [184], Sponge [185], Tuna [186], Abalone [187], Marine Snail [188], and so forth. The researchers used HPLC with different chromatographic columns and elution conditions to collect the narrow peaks; fractions showing remarkable activities were freeze-dried and further analysed to identify the amino acid composition and sequences, while HPLC also has some limitations, like chromatographic columns being expensive, elution composition containing organic solvent and being environmentally unfriendly, and so forth.

In recent years, HPLC is usually combined with qualitative equipment such as mass spectrometry (MS), and liquid chromatography followed by tandem mass spectrometric detection (LC–MS/MS) is the standard method for the characterization of peptide sequences [98], which has opened a new era in the structural elucidation of protein and peptides [189]; although this method is very precise and robust, it remains expensive and time-consuming [190]. In addition, electrospray ionization (ESI) and matrix-assisted laser desorption ionizations (MALDI) have appeared as important tools for protein identification and characterization [191]; matrix-assisted laser desorption/ionization time-of-flight (MALDI-TOF) mass spectrometric analysis is the backbone analysis for generating the peptide profiles of protein hydrolysates or semipurified fractions [179, 192], and so forth.

Besides, in order to alleviate insufficiencies, inadequacy, and disadvantages of the existing techniques, some researchers have developed new, rapid, specific, cost- and time-effective methods, such as high-performance liquid chromatography with evaporative light scattering detection (HPLC-ELSD), which can be used as an investigation tool for purification and quantitative measurements [193]. And as efficiency and speed of analysis have become of great importance in the field of bioanalysis, it is very important to increase the throughput and reduce the analysis costs; ultra-high-performance liquid chromatography-tandem mass spectrometry (UHPLC-MS/MS) analysis [194] and rapid resolution liquid chromatography-tandem mass spectrometry (RRLC–MS) [195] give new possibilities in this area.

In summary, there are increasing numbers of high efficiency and high resolution technologies for separation and purification. Thus, researchers should choose appropriate separation methods and media. Although some separation methods are still complex, time-consuming, and costly, scientists are committed to finding better methods to replace them, and the testing method would be more advanced.

## 5. Conclusion

Marine resources have been identified as excellent reservoirs for the extraction of potent functional bioactivities compounds. Therefore, large numbers of bioactive peptides have been isolated from marine organisms and display strong antioxidant, antihypertension, antimicrobial, anticoagulant and antidiabetic activities, and so forth. However, thus far, a limited number of bioactive peptides have been identified from marine organisms; most of the marine organisms with special biological activity are not yet been found. Thus, the existing manufacturing techniques need further improvement in order to find out more marine bioactive peptides. Furthermore, the most important problem is applying the bioactive peptides to human health and nutrition, because most of the researches stay in the stages of* in vitro* experiment or animal experiment due to the time-consumption and cost problems. In addition, the purification techniques developing rapidly in recent years and various media have been researched and improved; however, low yield and high cost are still a limiting factor. As a result, on the basis of guaranteeing the high selectivity and high resolution, how to improve the separation and purification technology is a difficult and significant task.

## Figures and Tables

**Table 1:** Biological activity associated with protein hydrolysates and peptides from marine organisms.

Common name	Scientific name	Origin	Biological activity	Peptide(s) sequence	Reference
Hoki	*Johnius belengerii*	Frame	Antioxidant	ESTVPERTHPACPDFN	[9]
Mackerel	*Pneumatophorus japonicus*	Muscle	Antioxidant	—	[10]
Mussel	*Perna canaliculus*	Muscle	Antioxidant	KGYSSYICDK, SSYCIVKICDK	[11]
Croaker	*Otolithes ruber*	Muscle	Antioxidant	KTFCGRH-	[12]
Tuna		Backbone	Antioxidant	VKAGFAWTANQQLS	[13]
Prawn	*Penaeus japonicus*	Muscle	Antioxidant	IKK, FKK, FIKK	[14]
Sardinelle	*Sardinella aurita*	Muscle	Antioxidant	LHT, LAAL, GGG, GAH, GATA, PHTL, GALAAH	[16]
Jumbo Squid	*Dosidicus gigas*	Skin	Antioxidant	FDSGPAGVL, NGPLQAGQPGER	[19]
Sea Cucumber	*Acaudina molpadioides*	Whole body	ACE inhibitory	MEGAQEAQGD	[31]
Tuna	—	Frame	Antihypertensive	GDLGKTTTVSNWSPPKYKDTP	[32]
Sole	*Limanda aspera*	Frame	Antihypertensive	MIFPGAGGPEL	[33]
Blue Mussel	*Mytilus edulis*	Whole body	ACE inhibitory	EVMAGNLYPG	[34]
Shrimp	—	Fermented product	ACE inhibitory	SV, IF, WP	[35]
Shrimp	*Plesionika izumiae Omori*	Whole shrimp	Antihypertensive	VWYHT, VW	[36]
Alaska Pollack	*Theragra chalcogramma*	Frame	ACE inhibitory	FGASTRGA	[37]
Alaska Pollack	*Theragra chalcogramma*	skin	ACE inhibitory	GPL, GPM	[38]
Tuna	—	Frame	ACE inhibitory	GDLGKTTTVSNWSPPKYKDTP	[32]
Shark	—	Meat	ACE inhibitory	CF, EY, MF, FE	[39]
Oyster	*Crassostrea gigas*	Muscle	Anti-HIV	LLEYSL, LLEYSI	[40]
Yellow Catfish	*Pelteobagrus fulvidraco*	Skin Mucus	Antimicrobial	GKLNLFLSRLEILKLFVGAL	[41]
Marine Snail	*Cenchritis muricatus*	Whole body	Antifungal	SRSELIVHQR	[42]
Hoki	*Johnius belengerii*	Frame	Ca-binding	VLSGGTTMYASLYAE	[43]
Alaska Pollack	Theragra chalcogramma	Backbone	Ca-binding	VLSGGTTMAMYTLV	[44]
Yellowfin Sole	*Limanda aspera*	Frame	Anticoagulant	TDGSEDYGILEIDSR	[45]
Spirulina Maxima	—	Whole body	Antiatherosclerotic	LDAVNR, MMLDF	[46]
Blue Mussel	*Mytilus edulis*	Whole body	Anticoagulant	EADIDGDGQVNYEEFVAMMTSK	[47]
Oyster	*Crassostrea gigas*	Muscle	Antitumour	—	[48]
Tuna	*Thunnus tonggol*	Muscle	Antiproliferative	LPHVLTPEAGAT, PTAEGGVYMVT	[49]
Pacific Whiting	*Merluccius productus*	Whole body	Immunomodulatory	—	[50]
Algae	*Pyropia yezoensis*	Whole body	Anti-inflammatory	—	[51]
Salmo	*Oncorhynchus keta*	Skin	Antidiabetic	—	[52]
Brown Shrimp	*Penaeus aztecus*	Head	Antiobesity	—	[53]

## Abbreviations

ROS: Reactive oxygen species
BHA: Butylated hydroxyanisole
BHT: Butylated hydroxytoluene
CAA: Cellular antioxidant activity
ACE: Angiotensin-I-converting enzyme
HPLC: High-performance liquid chromatography
CE: Capillary electrophoresis
AMPs: Antimicrobial peptides
H_2_SO_4_: Sulfuric acid
HNO_3_: Nitric acid
HCl: Hydrochloric acid
H_3_PO_4_: Phosphoric acid
RP-HPLC: Reversed-phase high-performance liquid chromatography
MWCO: Molecular weight cut-off
GFC: Gel filtration chromatography
IEX: Ion exchange chromatography
MS: Mass spectrometry
ESI: Electrospray ionization
MALDI: Matrix-assisted laser desorption ionizations
LC–MS/MS: Liquid chromatography followed by tandem mass spectrometric detection
MALDI-TOF: Matrix-assisted laser desorption/ionization time-of-flight.
